# A Photoelectrochemical Sensor Based on DNA Bio-Dots-Induced Aggregation of AuNPs for Methionine Detection

**DOI:** 10.3390/molecules28237740

**Published:** 2023-11-24

**Authors:** Chen Luo, Xiaoxiao Chen, Pu Li, Chaobiao Huang

**Affiliations:** 1 Xingzhi College, Zhejiang Normal University, Lanxi 321100, China; 202120200767@zjnu.edu.cn; 2 College of Chemistry and Materials Science, Zhejiang Normal University, Jinhua 321004, China; 201920200478@zjnu.edu.cn (X.C.); 202020200588@zjnu.edu.cn (P.L.)

**Keywords:** electroanalytical methods, gold nanoparticles, DNA bio-dots, methionine, fluorescence resonance energy transfer

## Abstract

Based on DNA bio-dots-induced aggregation of gold nanoparticles (AuNPs), a methionine (Met) photoelectrochemical (PEC) sensor with CS–GSH–CuNCs/TiO_2_ NPs as the photoelectric conversion element and AuNPs as the specific recognition element was constructed. First, a TiO_2_ NPs/ITO electrode and CS–GSH–CuNCs were prepared, and then the CS–GSH–CuNCs/TiO_2_ NPs/ITO photosensitive electrode was obtained by self-assembly. Next, DNA bio-dots were modified to the upper surface of the electrode using a coupling reaction to assemble the DNA bio-dots/CS–GSH–CuNCs/TiO_2_ NPs electrode. Amino-rich DNA bio-dots were used to induce the aggregation of AuNPs on the electrode surface via Au–N interactions and prepare the AuNPs/DNA bio-dots/CS–GSH–CuNCs/TiO_2_ NPs electrode. Due to the fluorescence resonance energy transfer (FRET) between CS–GSH–CuNCs and AuNPs, the complexation chance of electron-hole (e^−^-h^+^) pair in CS–GSH–CuNCs increased, which, in turn, led to a decrease in photocurrent intensity. When Met was present, AuNPs aggregated on the electrode surface were shed and bound to Met since the Au–S interaction is stronger than the Au–N interaction, resulting in the recovery of the photocurrent signal. Under optimal conditions, the photocurrent intensity of the PEC sensor showed good linearity with the logarithm of Met concentration in the range of 25.0 nmol/L–10.0 μmol/L with the limit of detection (LOD) of 5.1 nmol/L (S/N = 3, n = 10).

## 1. Introduction

Methionine (Met) is an essential amino acid that plays a vital role in cell metabolism, immune cell production, normal nerve function, growth and development, and many other tissue functions [1,2,3]. At the same time, Met plays an important role in human physiological and pathological processes. For example, Met is the main source of methyl in biomethylation, a precursor of many metabolites such as S-adenosyl methionine, and a major source of dietary sulfur. Increasing the production of lecithin in the liver facilitates the reduction in cholesterol levels and maintains normal cell growth [4,5,6,7,8]. Studies have demonstrated that a deficiency of Met would result in several diseases, such as AIDS and Parkinson’s disease [7]. Due to the clinical and physiological significance of Met [9], several methods have been established to detect Met, such as capillary electrophoresis [10,11], high-performance liquid chromatography [12,13], chemiluminescence [14], electrochemistry [15,16,17], fluorescence [18,19], and colorimetric methods [20,21]. However, these methods still have disadvantages such as high cost, a time-consuming process, and low sensitivity [22,23,24].

The photoelectrochemical (PEC) detection method is an emerging and rapidly developing assay method that has received a lot of attention in recent years. Many PEC sensors have been developed for the detection of chemical/biological molecules such as immune molecules, proteins, and some small molecules due to their high sensitivity, time-saving process, and ease of operation [25,26,27]. DNA-driven bio-dots (DNA bio-dots) have some properties such as low cytotoxicity, excellent dispersion, stable fluorescence, and good biocompatibility [28,29]. In addition, since DNA bio-dots retain the main conformation of DNA, they can act not only as luminescent elements but also as recognition elements, which makes them promising for applications in biosensing and bioimaging [9,30,31,32,33]. Currently, DNA bio-dots are mostly used in fluorescence detection and have extremely limited applications in PEC sensing. Therefore, it is imperative to expand the application of DNA bio-dots in PEC sensing.

**Scheme 1:**
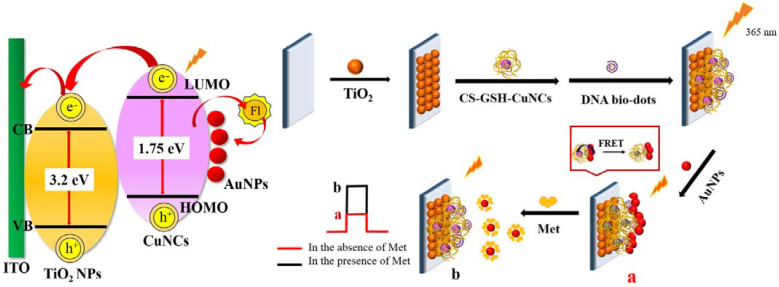
Construction process and mechanism of PEC sensor for Met detection.

The detection performance of PEC sensors is closely related to the modified photosensitive materials. It is demonstrated that chitosan-coated and glutathione-protected copper nanocluster/titanium dioxide nanoparticles (CS–GSH–CuNCs/TiO_2_ NPs) are a good photosensitive composite [34]. Photoexcitation at a wavelength of 365 nm causes the electrons in a copper nanocluster to move from the highest occupied molecular orbital (HOMO) to the lowest unoccupied molecular orbital (LUMO) [35]. TiO_2_ NPs are semiconductors with a wider energy band gap (~3.2 eV) than that of the CS–GSH–CuNCs (~1.75 eV), and the LUMO level of CS–GSH–CuNCs (~3.7 eV) is higher than the conduction band level of TiO_2_ NPs (~4.2 eV). Hence, as shown in Scheme 1, the photogenerated electrons were moved to the conduction band of TiO_2_, and the electron hole (e^−^-h^+^) was spatially separated.

In addition, it has been shown that Met can effectively inhibit the aggregation-inducing effect of DNA bio-dots on gold nanoparticles (AuNPs) [9]. Based on the above, a PEC sensor for detecting Met was constructed, as shown in Scheme 1. First, CS–GSH–CuNCs/TiO_2_ NPs photosensitive electrodes were prepared by sintering and self-assembly, and then DNA bio-dots were modified to the upper surface of the electrode using a coupling reaction to obtain the DNA bio-dots/CS–GSH–CuNCs/TiO_2_ NPs electrode. The surface of DNA bio-dots is rich in amine groups, and DNA bio-dots with a low negative charge would replace the weakly adsorbed citrate ions on the surface of AuNPs. The strong Au–N interactions reduce the electrostatic repulsion between neighboring AuNPs, so AuNPs would aggregate on the electrode surface [36,37,38,39]. Finally, the AuNPs/DNA bio-dots/CS–GSH–CuNCs/TiO_2_ NPs electrode was prepared. Because of the fluorescence resonance energy transfer (FRET) between AuNPs and CS–GSH–CuNCs, the electron hole (e^−^-h^+^) pair complexation chance in CS–GSH–CuNCs increased, which resulted in the decrease in photocurrent. However, AuNPs aggregated on the electrode surface fall off and bind to Met in the presence of Met. This is because the Au–S interaction was stronger than the Au–N interaction, which, in turn, led to the recovery of the photocurrent signal.

## 2. Results and Discussion

### 2.1. Spectroscopic Properties of AuNPs, DNA Bio-Dots, and CS–GSH–CuNCs

**Figure 1:**
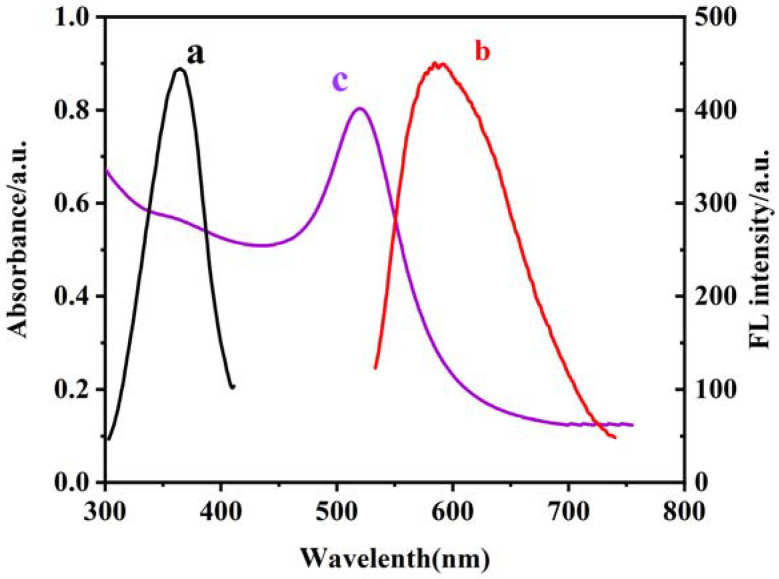
Excitation spectra (a) and emission spectra (b) of CS–GSH–CuNCs and UV absorption spectra of AuNPs (c). The scanning speed is 150 nm/s and the slit is 10 nm.

The curves a, b, and c in Figure 1 show the excitation and emission spectra of CS–GSH–CuNCs and the UV-vis absorption spectra of AuNPs, respectively. As the results show, the maximum excitation and emission wavelengths were 365 nm and 586 nm, and the absorption peak of AuNPs was located at 518 nm with the continuous absorption in the visible region. An obvious spectral overlap between the emission band of CS–GSH–CuNCs and the absorption band of AuNPs was observed, which indicates the fluorescence resonance energy transfer (FRET) effect between CS–GSH–CuNCs and AuNPs. In the subsequent experiment, the excitation wavelength was selected to 365 nm.

### 2.2. Characterization of Materials

#### 2.2.1. SEM of Different Modified Electrodes

**Figure 2:**
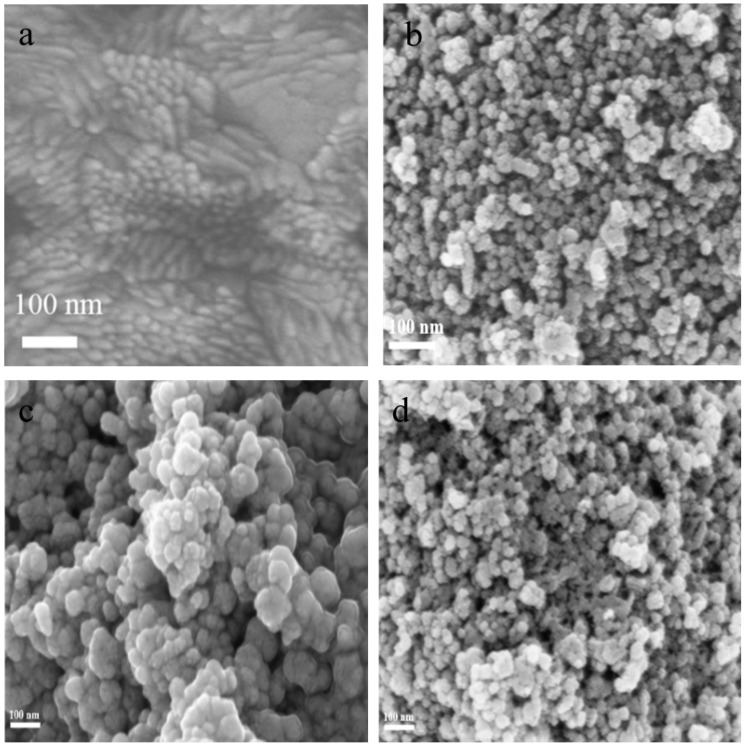
SEM image of (**a**) bare ITO electrode, (**b**) TiO_2_ NPs/ITO, (**c**) CS–GSH–CuNCs/TiO_2_ NPs/ITO, and (**d**) DNA bio-dots/CS–GSH–CuNCs/TiO_2_ NPs/ITO.

Figure 2 shows the SEM image of different modified electrodes. Figure 2a shows the SEM of the bare ITO electrode, which is smooth. As shown in Figure 2b, it could be observed that the TiO_2_ NPs had a typical spherical nanoparticle morphology, and TiO_2_ NPs were covered with stripes of CS–GSH–CuNCs particles in the outer layer, as seen in Figure 2c. As shown in Figure 2d, compared with the CS–GSH–CuNCs/TiO_2_ NPs/ITO electrode, the significantly increased voids and surface roughness of the DNA bio-dots/CS–GSH–CuNCs/TiO_2_ NPs/ITO electrode indicated that DNA bio-dots have been modified to the surface of the CS–GSH–CuNCs/TiO_2_ NPs/ITO electrode.

#### 2.2.2. XPS of Different Modified Electrodes

**Figure 3:**
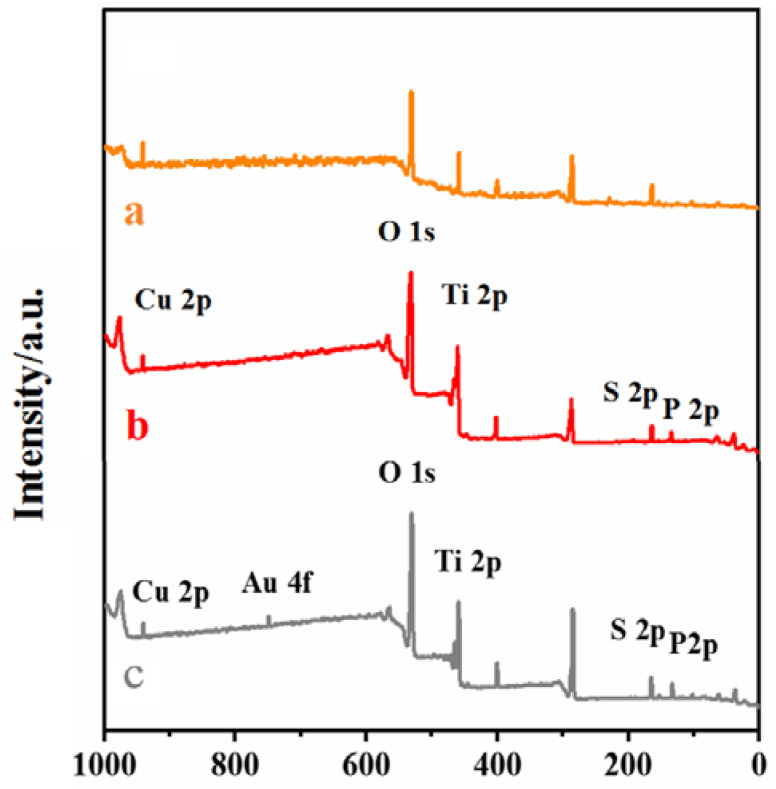
XPS scanning spectra: (a) CS–GSH–CuNCs/TiO_2_ NPs, (b) DNA bio-dots/CS–GSH–CuNCs/TiO_2_ NPs, (c) AuNPs/DNA bio-dots/CS–GSH–CuNCs/TiO_2_ NPs.

As shown in Figure 3, curves a, b, and c are XPS spectra of CS–GSH–CuNCs/TiO_2_ NPs/ITO electrode, DNA bio-dots/CS–GSH–CuNCs/TiO_2_ NPs/ITO electrode, and AuNPs/DNA bio-dots/CS–GSH–CuNCs/TiO_2_ NPs/ITO, respectively, which can be obviously observed that Ti, O, Cu, and S elements are present. This indicates that CS–GSH–CuNCs successfully self-assembled on the surface of the TiO_2_ NPs/ITO electrode. And compared with curve a, the P element attributed to ${\mathrm{PO}}_{4}^{1-}$ of DNA bio-dots could be additionally observed in curve b [40]. In curve c, the presence of the Au element can be obviously found. The above results indicated that DNA bio-dots and AuNPs have been successfully modified to the surface of the TiO_2_ NPs/ITO electrode and the AuNPs/DNA bio-dots/CS–GSH–CuNCs/TiO_2_ NPs/ITO electrode was successfully prepared.

**Figure 4:**
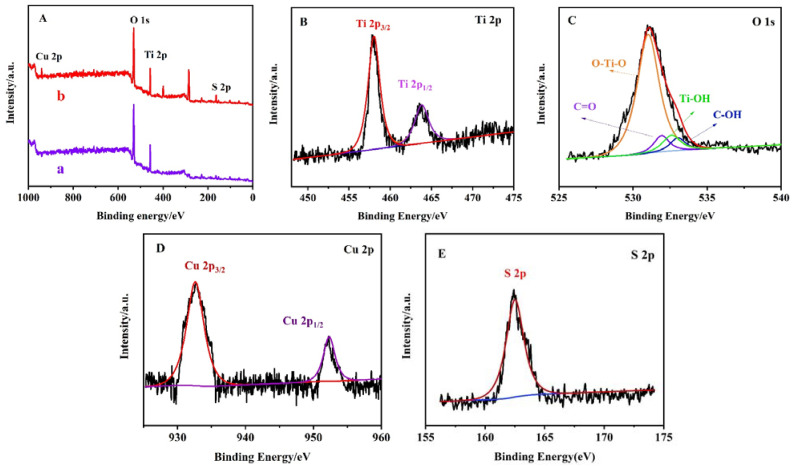
(**A**) The full-scan XPS spectra of CS–GSH–CuNCs/TiO_2_ (a) NPs and TiO_2_ NPs (b). XPS curves of CS–GSH–CuNCs/TiO_2_ NPs: (**B**) Ti 2p, (**C**) O 1s, (**D**) Cu 2p and (**E**) S 2p.

The XPS spectra further revealed the valence state and composition of the elements. As shown in Figure 4A, curves a and b are the full-scan XPS spectra of the TiO_2_ NPs/ITO electrode and CS–GSH–CuNCs/TiO_2_ NPs/ITO electrode, which can find Ti and O elements and Ti, O, Cu, and S elements, respectively. Figure 4B depicts the spectrum of Ti 2p with characteristic peaks at 458.52 and 464.35 eV, corresponding to Ti 2p_3/2_ and Ti 2p_1/2_, indicating that the Ti element is in the complete oxidation state of Ti^4+^. Figure 4C shows the XPS spectra of O 1s, in which the characteristic peaks of O 1s with the banding energies of 529.47 and 531.12 eV were attributed to the lattice oxygen Ti-O and surface hydroxyl oxygen Ti-OH in anatases TiO_2_ NPs, respectively. This means that oxygen existed in the form of O^2−^. Two peaks at 531.82 and 533.13 eV were attributed to C=O and C-OH. Figure 4D shows the Cu 2p XPS spectra of CS–GSH–CuNCs. The peaks with banding energies of 932.65 and 953.15 eV were ascribed to 2p_3/2_ and 2p_1/2_ electrons of Cu(0), and the lacking peak at 942.06 eV implied the absence of Cu^2+^. The difference between the 2p_3/2_ binding energy of Cu(0) and Cu^+^ differs by only 0.10 eV. Hence, in general, the oxidation state of the Cu atom in CS–GSH–CuNCs is between zero and one. Furthermore, Figure 4E shows the binding energy peak of the S 2p_3/2_, which obviously indicates that the -SH is bound to Cu through Cu–S bond formation. To sum up, the chemical states and elemental compositions from the above spectra further indicate that the CS–GSH–CuNCs/TiO_2_ NPs/ITO electrode were prepared as expected.

#### 2.2.3. EIS of Different Modified Electrodes

**Figure 5:**
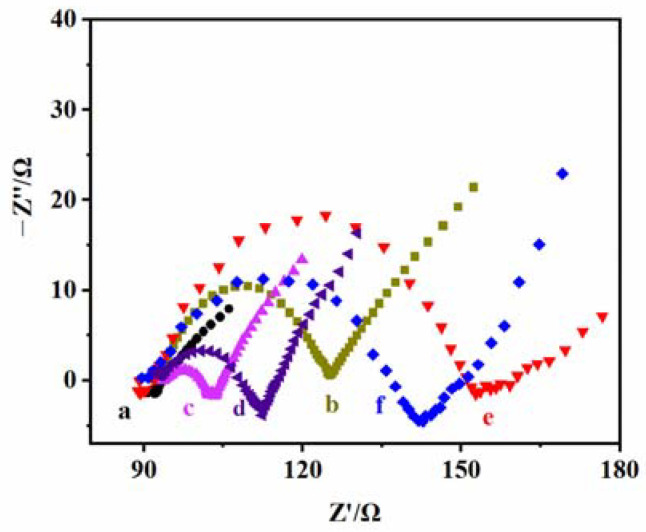
EIS of (a) the bare ITO electrode, (b) TiO_2_ NPs/ITO electrode, (c) CS–GSH–CuNCs/TiO_2_ NPs/ITO electrode, (d) DNA bio-dots/CS–GSH–CuNCs/TiO_2_ NPs/ITO electrode, (e) AuNPs/DNA bio-dots/CS–GSH–CuNCs/TiO_2_ NPs/ITO electrode, and (f) AuNPs/DNA bio-dots/CS–GSH–CuNCs/TiO_2_ NPs/ITO electrode incubated + 0.10 μmol/L Met. (Electrolyte: 5.0 mmol/L K_3_Fe(CN)_6_/K_4_Fe(CN)_6_ (1:1) containing 0.1 mol/L KCl, detection conditions: sine wave amplitude: 50 mV; scanning frequency: 0.1–105 Hz).

Figure 5 shows the EIS of the electrodes at different modification stages, where curve a is the electrical impedance curve of the bare ITO electrode. It indicates that its electrical impedance value is very small and its conductive performance is excellent. Curve b is the electrical impedance curve of the TiO_2_ NPs/ITO electrode. It can be observed that the impedance of the electrode increases after TiO_2_ NPs are loaded, which is because of the poor electrical conductivity of TiO_2_ NPs. Curve c is the electrical impedance curve of the CS–GSH–CuNCs/TiO_2_ NPs/ITO electrode. Since the CS wrapped in the outer layer of the copper nanoclusters has a positive charge, the electron transfer of the negatively charged probe ([Fe-(CN)_6_)]^3−/4−^) is effectively improved [41], thus significantly reducing the impedance of the electrode. Curve d is the electrical impedance curve of the DNA-bio-dots/CS–GSH–CuNCs/TiO_2_ NPs/ITO electrode. With the introduction of DNA bio-dots, the conductivity of the electrode decreases, and the impedance increases. Curve e is the electrical impedance curve of the AuNPs/DNA bio-dots/CS–GSH–CuNCs/TiO_2_ NPs/ITO electrode. The electrical impedance of the electrode increases to maximum. This is because AuNPs accumulate on the electrode surface, resulting in an increased e^-^-h^+^ recombination probability in CS–GSH–CuNCs and a reduced transfer of photogenerated electrons to the electrode [34]. However, when Met is present, AuNPs gathered on the electrode surface fall off and bind with Met because the interaction between Au and S is stronger than the interaction between Au and N [37,38,42], and the electrode conductivity is enhanced and the impedance is reduced (curve f). The above results prove the successful construction of the PEC sensor.

### 2.3. Effect of Incubation Time of DNA Bio-Dots and AuNPs on PEC Sensor

**Figure 6:**
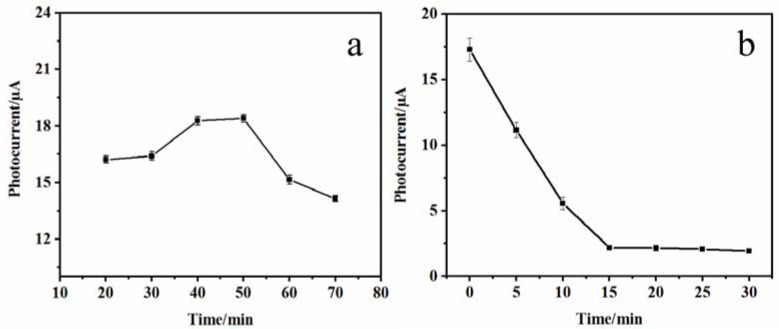
Photocurrent signal with incubation time of DNA bio-dots (**a**) and AuNPs (**b**).

The incubation time of DNA bio-dots was optimized for improving the detection performance of the PEC sensor. Figure 6a shows the variation curve of the photocurrent signal of the CS–GSH–CuNCs/TiO_2_ NPs/ITO electrode with the incubation time in the NDA bio-dots solution. This indicates that when the incubation time is less than 40 min, the photocurrent signal increases with the extension of the incubation time. This may be due to the fact that a small number of DNA bio-dots are excited at the same time as the CS–GSH–CuNCs are excited, or the modification of DNA bio-dots and the photogenerated electrons of CS–GSH–CuNCs were further transferred to the TiO_2_ NPs conduction band. When the incubation time was 40–50 min, the photocurrent signal remained basically stable. However, when the incubation time exceeded 50 min, the photocurrent signal decreased, which may be due to the increase in steric hindrance at the electrode interface and the reduction in excitation efficiency of CS–GSH–CuNCs due to the excessive loading of DNA bio-dots. Therefore, 40 min was selected as the optimal incubation time for DNA bio-dots in subsequent experiments.

In addition, the optimal incubation time of AuNPs was also chosen. As shown in Figure 6b, the photocurrent signal of the AuNPs/DNA bio-dots/CS–GSH–CuNCs/TiO_2_ NPs/ITO electrode gradually decreased with the extension of incubation time. When the incubation time exceeded 15 min, the photocurrent signal basically reached the minimum and stabilized. Therefore, 15 min was chosen as the optimal time for the incubation of the electrode with AuNPs in the subsequent experiment. 

### 2.4. Electrochemical Properties

#### 2.4.1. PEC Behavior of the Sensor

**Figure 7:**
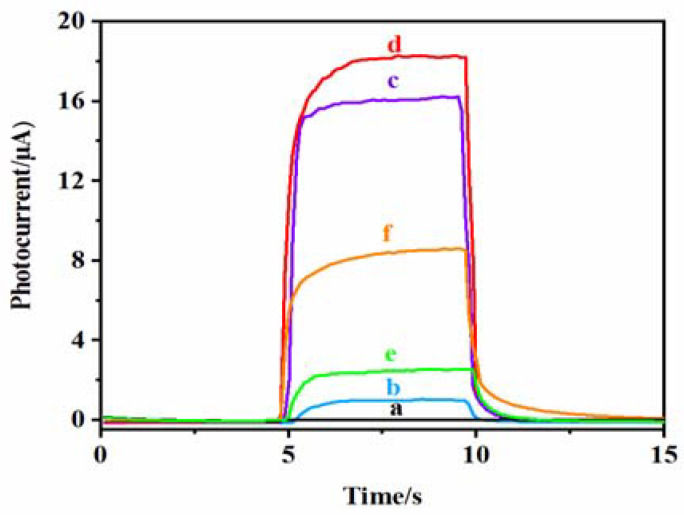
Photocurrent response curve of (a) the bare ITO electrode, (b) TiO_2_ NPs/ITO electrode, (c) CS–GSH–CuNCs/TiO_2_ NPs/ITO electrode, (d) DNA bio-dots/CS–GSH–CuNCs/TiO_2_ NPs/ITO electrode, (e) AuNPs/DNA bio-dots/CS–GSH–CuNCs/TiO_2_ NPs/ITO electrode, and (f) AuNPs/DNA bio-dots/CS–GSH–CuNCs/TiO_2_ NPs/ITO electrode immersed in PBS buffer containing 0.10 μmol/L Met. (The PEC tests were obtained in PBS buffer (0.1 mol/L, pH 7.4) with 365 nm excitation light and 0.0 V applied voltage. Reference electrode is Ag/AgCl electrode.

Figure 7 shows the photocurrent response of each electrode at different modification stages. Curve a shows that the bare ITO electrode has almost no photocurrent response. Curve b is the photocurrent response of TiO_2_ NPs/ITO, which increases slightly because TiO_2_ NPs are a wide energy band gap (~3.2 eV) semiconductor that can only absorb UV light, and the photoelectric conversion efficiency is low [43]. The photocurrent signal of the DNA CS–GSH–CuNCs/TiO_2_ NPs/ITO electrode is enhanced (curve c), which is attributed to the LUMO level of CS–GSH–CuNCs being higher than the conduction band level of TiO_2_ NPs. When CS–GSH–CuNCs are excited by 365 nm, the photogenerated electrons in LUMO are moved to the conduction band of TiO_2_ NPs, effectively reducing the recombination probability of e^−^-h^+^ [44,45]. After modifying DNA bio-dots on the surface of the CS–GSH–CuNCs/TiO_2_ NPs/ITO electrode, the photocurrent signal continued to be enhanced (Curve d). This may be because a small number of DNA bio-dots were also excited at the same time when CS–GSH–CuNCs were excited, or because the modification of DNA bio-dots led to a further increase in the transfer of photogenerated electrons from the LUMO of CS–GSH–CuNCs to the conduction band of TiO_2_ NPs. After incubation with AuNPs, the photocurrent intensity of the AuNPs/CS–GSH–CuNCs/TiO_2_ NPs/ITO electrode decreased significantly due to the FRET effect between AuNPs and CS–GSH–CuNCs (Curve e). When Met is present in the solution, the photocurrent signal of the electrode is restored (curve f), because Met can effectively prevent AuNPs from aggregation and cause aggregated AuNPs to fall off the electrode surface [9].

**Figure 8:**
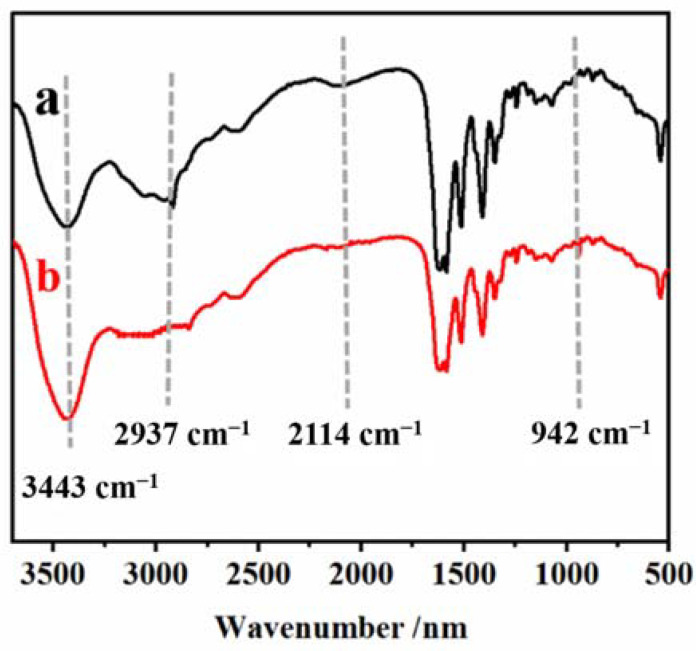
The FTIR spectra of (a) Met and (b) the remnant of PEC test solution which was conducted using rotary evaporation. The samples were fully dried using rotating evaporation. The samples were mixed, dried, and pressed with KBr at a ratio of about 1:100, respectively.

Curves a and b in Figure 8, respectively, are the infrared spectra of Met and the remnant of the PEC test solution which conducted rotary evaporation. The characteristic peak of ${\mathrm{NH}}_{3}^{+}$ at 2937 cm^−1^ confirms the presence of zwitterionic Met [39,46]. In curve b, the spectral band at 2937 cm^−1^ disappeared, the spectral band at 3443 cm^−1^ enhanced, and the ${\mathrm{COO}}^{-}$ -${\mathrm{HNH}}_{2}^{+}$ band at 2114 cm^−1^ disappeared, which was attributed to the AuNPs falling off the electrode surface combined with Met, triggering Met reorientation. This results in the formation of new Met configurations [9]. In addition, the characteristic absorption peak of Au–S symmetric stretching in the AuNPs–Met complex at 942 cm^−1^ can also be observed in curve b [39]. These results confirmed the existence of the AuNPs–Met complex and further demonstrated that Met could effectively inhibit the aggregation of AuNPs induced by DNA-bio-dots on the electrode surface.

#### 2.4.2. Detection Performance of PEC Sensor for Met

**Figure 9:**
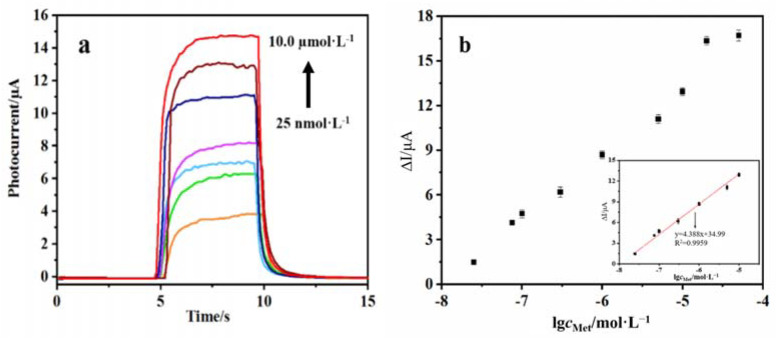
(**a**) Photocurrent intensity of PEC sensor under different concentrations of Met (0.025, 0.075, 0.10, 0.30, 1.00, 5.00, 10.0 µmol/L), (**b**) calibration curve where ΔI = I − I_0_; I and I_0_ are the photocurrent intensity in the presence and absence of Met.

Under optimal conditions, AuNPs/DNA bio-dots/CS–GSH–CuNCs/TiO_2_ NPs/ITO electrodes were immersed in an incubation solution containing different concentrations of Met, and the photocurrent signal was recorded. The results showed that the recovery degree of the photocurrent signal of the PEC sensor was related to the Met concentration. As shown in Figure 9a, the photocurrent intensity of the PEC sensor gradually increases with the increase in Met concentration. Figure 9b shows that the change in photocurrent intensity of the PEC sensor has a good linear relation with the logarithm of Met concentration within the range of 25.0 nmol/L to 10.0 μmol/L. The linear equation is ∆Ι(μA) = 4.388 lg*c*_Met_ + 34.99 (R^2^ = 0.9959), and the LOD of the PEC sensor is 5.1 nmol/L (S/N = 3, n = 10). Compared with other reported quantitative analysis methods (Table 1), the PEC sensor constructed in this work has a lower detection limit for Met, demonstrating its superiority.

#### 2.4.3. Selectivity, Stability, and Reproducibility of PEC Sensors

**Figure 10:**
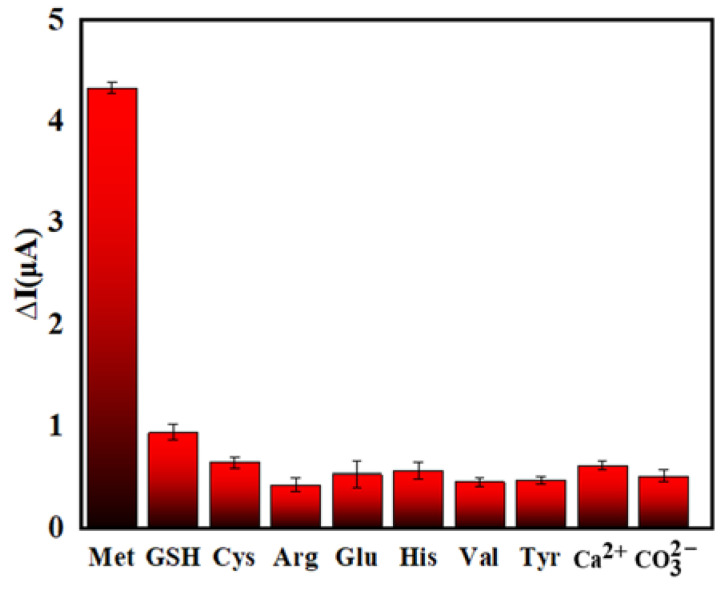
Selectivity of PEC sensor for Met detection (The concentration of interfering substances: 1.00 μmol/L, the concentration of Met: 0.10 μmol/L).

In order to explore the selectivity of the PEC sensor, a series of interfering substances (GSH, Cys, Arg, Glu, His, Val, Tyr, Ca^2+^, ${\mathrm{CO}}_{3}^{2-}$) was selected that may interfere with the detection of Met. Studies have shown that sulfhydryl compounds such as GSH and Cys can also inhibit the aggregation of AuNPs. Therefore, in order to eliminate the interference of these compounds, *N*-ethylmaleimide (NEM) was selected as a masking agent. The experimental results are shown in Figure 10. They demonstrate that the PEC sensor is essentially free from the interference of sulfhydryl compounds, such as GSH and Cys, when NEM is used. This suggests that the sensor has good selectivity for detecting Met.

**Figure 11:**
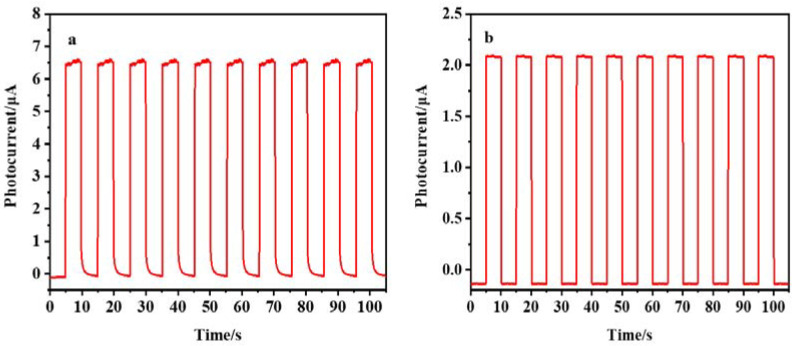
Photocurrent response curve of PEC sensor in the presence (**a**) and absence (**b**) of Met (0.10 µmol/L).

We also studied the stability and reproducibility of PEC sensors. Figure 11a,b respectively indicate that, under the best conditions, the photocurrent response curves are obtained by repeating the on/off lighting cycle every 5 s for 105 s when AuNPs/DNA bio-dots/CS–GSH–CuNCs/TiO_2_ NPs/ITO electrodes were present at 0.10 µmol/L (a) and 0.00 µmol/L Met (b). The results showed that the photocurrent intensity of the electrode had little change with the increase in illumination time and detection times, whether Met exists or not. In addition, the prepared AuNPs/DNA bio-dots/CS–GSH–CuNCs/TiO_2_ NPs/ITO electrode was stored in a refrigerator at 4 °C for 3 weeks, and then its photocurrent intensity was measured. The results show that the photocurrent intensity of the electrode only decreased by 1.7%, indicating that the sensor had good stability. Finally, in order to study the reproducibility of the PEC sensor, the photocurrent intensity of 9 parallel-prepared PEC sensors in the presence of 0.10 μmol/L Met was recorded under the same experimental conditions. The results show that the calculated RSD was about 3.0%, indicating that the PEC sensor had good reproducibility.

### 2.5. Actual Sample Analysis and Recovery Detection

Under optimal conditions, AuNPs/DNA bio-dots/CS–GSH–CuNCs/TiO2 NPs/ITO electrodes were immersed in urine samples containing various concentrations of Met (0.05, 0.30, and 1.00 μmol/L). And the results are shown in Table 2. There is no Met detected in urine samples, and the recovery rate was 97.4%–104.0%, indicating that the PEC sensor has a potential application prospect in the detection of Met in real samples. 

## 3. Materials and Methods

### 3.1. Reagents

Chitosan (CS), chloroauric acid (HAuCl_4_), glutathione (GSH), arginine (Arg), glutamate (Glu), histidine (His), valine (Val), tyrosine (Tyr), methionine (Met), cysteine (Cys), and low molecular weight double-stranded salmon DNA were acquired from Aladdin Co., Ltd. (Shanghai, China). Titanium dioxide (TiO_2_) was supplied by Adamas Reagent Co., Ltd. (Shanghai, China). Cu(NO_3_)_2_, KCl, NaH_2_PO_4_, Na_2_HPO_4_, NaCl, ethanol, acetic acid, acetone, sodium citrate, *N*-ethylmaleimide (NEM), and aqua regia (mixture of HCl and HNO_3_ at a 3:1 ratio) were acquired from Sinopharm Chemical Reagent Co., Ltd. (Shanghai, China). ITO electrodes (resistivity 6–8 Ω) were acquired from Huanan Xiangcheng Technology Co., Ltd. (Shengzhen, China). All reagents are analytically pure. The water used in this work was ultrapure water.

### 3.2. Instruments

Electrochemical impedance spectroscopy (EIS) was recorded using the Autolab multifunctional electrochemical workstation (PGSTAT302N, Merohm Autolab B.V, Utrecht, The Netherlands). Fluorescence spectra were recorded using an RF-6000 fluorescence spectrometer (Shimadzu, Kyoto, Japan). Fluorescence (FL) spectra were recorded with an RF-6000 fluorescence spectrometer (Shimadzu, Japan). UV-vis absorption spectra were recorded using an LZMBDA 950 spectrophotometer (PerkinElmer Inc., Waltham, MA, USA). The FT-IR spectra were recorded using a Nexus 670 infrared spectrometer (Reonicolai Inc., New York, NY, USA). X-ray photoelectron spectra were characterized using an X-ray photoelectron spectrometer (XPS, ESCALAB 250Xi, ThermoFisher Scientific, Waltham, MA, USA). The surface morphology was characterized using scanning electron microscopy (SEM) (JSM-6360, JEOL, Zaventem, Belgium). PEC detection was conducted in a CHI850D electrochemical workstation (CH Instruments, Shanghai, China).

### 3.3. Synthesis of DNA Bio-Dots, CS–GSH–CuNCs, and AuNPs

#### 3.3.1. Synthesis of DNA Bio-Dots

DNA bio-dots were prepared according to the method reported in the literature [28]. Under the protection of N_2_, low molecular double-stranded salmon DNA was dispersed in ultra-pure water that had been deoxidized with N_2_ for 15 min to prepared into DNA solution with a concentration of 1 mg/mL. The DNA solution was heated to 85 °C and stirred for 11 h. Then, it was rapidly added to acetone under ultrasonic treatment for 1 h. Finally, a well-dispersed DNA bio-dots solution was prepared using filtration with a 0.22 μm film and stored in a refrigerator at 4 °C for later use.

#### 3.3.2. Synthesis of CS–GSH–CuNCs

CS–GSH–CuNCs were synthesized according to the method reported in the literature [41]. An amount of 20 mg CS was dissolved in 20 mL (0.4%) acetic acid solution and then mixed with 20 mL 0.06 mmol/L GSH aqueous solution. An amount of 2 mL 0.1 mmol/L Cu(NO_3_)_2_ solution was added to an ice bath with intense agitation. After 1 min of reaction, the mixture was filtered, the filtrate obtained was centrifuged at 10,000 rpm for 20 min, and then the precipitation was re-dispersed in ultrapure water. Finally, the prepared CS–GSH–CuNCs solution was stored in the refrigerator at 4 °C for later use.

#### 3.3.3. Synthesis of AuNPs

AuNPs were prepared using the citric acid reduction method reported in the literature [48]. HAuCl_4_ (1 mL, 1 wt%) and 99 mL of water were added to a round-bottomed flask soaked in aqua regia (a mixture of concentrated HCl and concentrated HNO_3_ at a ratio of 3:1) for 1 h and thoroughly cleaned with deionized water. The solution was heated to reflux, and then quickly add sodium citrate solution (2.5 mL 1.0 wt%) under intense agitation.). The color of the solution turned to wine red, and continued to boil for 15 min until the color of the solution no longer changed. Finally, the heating was stopped, and the AuNPs solution was continuously stirred and cooled to room temperature. The prepared AUNPs solution was stored in a refrigerator at 4 °C away from light for later use.

### 3.4. The Assembly of AuNPs/DNA Bio-Dots/CS–GSH–CuNCs/TiO_2_ NPs/ITO Electrode

ITO plates were immersed in boiled 1.0 mol/L NaOH solution for 15 min. Then, ITO plates were ultrasonically cleaned with acetone, ethanol, and ultrapure water for 10 min each, followed by drying in an oven at 60 °C. An amount of 4 mg TiO_2_ was dispersed in 1 mL of ultrapure water using ultrasound, and 30 µL of the uniform suspension was dropped onto an ITO plate with a fixed area of 0.25 cm^2^. After air drying and sintering at 450 °C for 1.5 h, the TiO_2_ NPs/ITO electrode was cooled to room temperature and immersed in as-prepared CS–GSH–CuNCs solution for 70 min. Then, the prepared CS–GSH–CuNCs/TiO_2_ NPs/ITO electrode was incubated in 2 mL DNA bio-dots master solution for 40 min and washed with ultrapure water. The DNA bio-dots/CS–GSH–CuNCs/TiO_2_ NPs/ITO electrode was obtained. Then, it was incubated with 100 μL AuNPs in PBS (0.1 mol/L, pH 7.4) containing 50 mmol/L NaCl for 15 min. Finally, the AuNPs/DNA bio-dots/CS–GSH–CuNCs/TiO_2_ NPs/ITO electrode was obtained and stored at 4 °C for subsequent use. 

### 3.5. Detection

#### 3.5.1. Detection of Methionine

The prepared AuNPs/DNA bio-dots/CS–GSH–CuNCs/TiO_2_ NPs/ITO electrodes were immersed with different concentrations of Met/PBS solution and detected using the PEC system. The detection potential was set at 0.0 V and the excitation wavelength was 365 nm.

#### 3.5.2. Masking of Distractions

*N*-ethylmaleimide (NEM) is a thiol blocker based on the Michael addition reaction [49]. Considering the interference of sulfhydryl-containing compounds such as GSH and Cys, 0.20 mol/L NEM and 0.10 µmol/L Met were mixed with 1.00 µmol/L interfering substances (GSH, Cys, Arg, Glu, His, Val, Tyr, Ca^2+^, ${\mathrm{CO}}_{3}^{2-}$).

#### 3.5.3. Actual Samples Handling

Several urine samples provided by three healthy volunteers were tested to reveal the sensor’s detection performance in real samples. The urine samples were simply centrifuged and the supernatant was filtered by a 0.22 μm filter membrane.

## 4. Conclusions

Based on AuNPs/DNA bio-dots/CS–GSH–CuNCs/TiO_2_ NPs/ITO electrodes, a novel PEC sensor was proposed for the detection of Met. Obviously, compared with other methods, the PEC sensor has the advantages of high sensitivity, good selectivity, and low detection limit. More profoundly, the PEC sensor was successfully used for the detection of Met in urine, which indicates that the PEC biosensor has great application potential in the detection of Met in real samples.

## Figures and Tables

**Table 1:** Comparison with other analytical methods for the detection of Met.

Analytical Method	Linear Range (µmol/L)	Detection Limit (nmol/L)	Ref.
Electrochemical method	0.10–10.0	100	[16]
Colorimetric method	0.00–1.00	24.5	[19]
High-performance liquid chromatography	2.00–60.0	1.0	[13]
Chemiluminescence	0.10–10.0	30.0	[14]
Fluorimetry	2.51–25.12	1000	[47]
Photoelectrochemistry	0.025–10.0	5.1	This work

**Table 2:** Detection and recovery of Met in actual samples.

Sample Entry	Found (µmol/L) $(\bar{x}$ ± SD, n = 3)	Added (µmol/L)	Detected (µmol/L) $(\bar{x}$ ± SD, n = 3)	Recovery (%) $(\bar{x}$ ± SD, n = 3)
1	0.00	0.050	0.052 ± 0.002	104.0 ± 3.8
2	0.00	0.300	0.292 ± 0.007	97.4 ± 2.3
3	0.00	1.000	1.036 ± 0.012	103.6 ± 1.6

## Data Availability

Data are contained within the article.
